# Tumor-Free Resection Margin Distance in the Surgical Treatment of Node-Negative Squamous Cell Cancer of the Vulva Has No Impact on Survival: Analysis of a Large Patient Cohort in a Tertiary Care Center

**DOI:** 10.3390/cancers15164110

**Published:** 2023-08-15

**Authors:** Florin Andrei Taran, Jana Pasternak, Annette Staebler, Annika Rohner, Felix Neis, Tobias Engler, Ernst Oberlechner, Birgitt Schönfisch, Ingolf Juhasz-Böss, Andreas Daniel Hartkopf, Sara Brucker, Christina Barbara Walter

**Affiliations:** 1 Department of Obstetrics and Gynecology, Medical Center, University of Freiburg, 79106 Freiburg, Germany; 2 Department of Women’s Health, Tuebingen University Hospital, 72076 Tuebingen, Germany; 3 Institute of Pathology and Neuropathology, Tuebingen University Hospital, 72076 Tuebingen, Germany

**Keywords:** vulvar cancer, resection margin distance, recurrence, survival

## Abstract

**Simple Summary:**

Squamous cell cancer (SCC) of the vulva is one of the rare gynecological malignancies, and surgery is the initial treatment of choice for early-stage vulvar cancer. International guidelines offer conflicting recommendations regarding surgical and pathological tumor-free resection margins for SCC of the vulva. The aim of this study was therefore to analyze the node-negative patients with SCC of the vulva treated with surgery alone at the Department of Women’s Health, University Clinic, Tuebingen, Germany with regard to the achieved marginal distance and its impact on prognosis. The present study observed no significant impact of pathological tumor-free resection margin distance following surgery in patients with node-negative SCC of the vulva on disease-free and overall survival.

**Abstract:**

Background: The aim of this study was to evaluate the impact of pathological tumor-free margin distance on survival in SCC patients treated with surgery alone. Methods: This retrospective study included 128 patients with node-negative disease that received no adjuvant treatment. Disease-free and overall survival were analyzed according to pathological tumor-free margin distance. Results: The patients were subclassified into three resection margin category groups: “1 to 3 mm” (n = 42), “>3 to 8 mm” (n = 47) or “>8 mm” (n = 39). Thirty-nine of the 128 patients (30.5%) developed recurrent disease. Median follow-up for disease-free survival (DFS) was 6.49 years (95% CI 5.16 years; 7.62 years), and median follow-up for overall survival (OS) was 6.29 years (95% CI 5.45 years; 7.33 years). The 5-year DFS rate was 0.70 (95% CI: 0.62–0.79), and the 5-year OS rate was 0.79 (95% CI: 0.71–0.87). Regarding the survival outcome, there were no independent significant differences in either disease-free survival (DFS) (*p* = 0.300) or overall survival (*p* = 1.000) among patients within the three tumor-free resection margin categories. Multivariate analyses did not show any statistically significant association between tumor-free resection margin distance and recurrent disease or death, either when analyzed as a categorical variable or when analyzed as a continuous variable. Conclusion: The present study did not show a significant impact of pathological tumor-free resection margin distance following surgery in patients with node-negative SCC of the vulva (that did not receive adjuvant treatment) on disease-free and overall survival.

## 1. Introduction

Squamous cell carcinoma (SCC) of the vulva is one of the rare gynecological malignancies. In 2019, 3293 new cases of vulvar cancer occurred in Germany [1]. Overall, vulvar cancer is a disease that tends to afflict older women, with a median age of onset of 73 years and most cases being diagnosed at an early stage, resulting in a 5-year survival rate of 73% [1]. SCC of the vulva is caused by two main pathways: oncogenic human papillomavirus (HPV) infection and independent HPV infection. HPV-associated SCC occurs at a younger age compared to non-HPV-associated SCC [2].

Surgery is the initial treatment of choice for early-stage vulvar cancer [3]. Radical local excision and inguinofemoral sentinel lymphadenectomy, or the removal of the tumor with an adequate margin, has become a common form of vulvar surgery, replacing radical vulvectomy with systematic inguinofemoral lymphadenectomy [3,4,5,6,7]. In the case of early-stage vulvar cancer with high-risk features, radiation therapy or combined radiochemotherapy must be considered [5,8,9].

However, the surgical procedure, in particular, sometimes faces surgeons with great challenges, especially due to the anatomical proximity to relevant structures (clitoris, urethra, introitus vaginae and anus). The extent of the clinical and histological resection margin is still a major point of discussion in the current literature [10,11,12,13]. Reconstruction and defect coverage also require a high level of surgical expertise, especially with regard to wound healing and patient morbidity [14]. Although the significance of the resection margin and its extent are ultimately not completely clear, a narrow resection margin for the patients concerned means that a second resection or further adjuvant treatment is discussed, combined with renewed morbidity [13].

International guidelines offer conflicting recommendations regarding surgical and pathological tumor-free resection margins for SCC of the vulva [15,16,17]. The German guideline concludes that an evidence-based cutoff for tumor-free resection margins cannot be defined; however, based on expert consensus, a pathological tumor-free margin of 3 mm should be the objective [17]. The ESGO guideline advises a surgical resection margin of at least 1 cm, with narrower margins being acceptable when the objective is preservation of the function of the clitoris, urethra and anus [16]. Additionally, the NCCN guideline recommends a surgical margin of at least 1 cm at primary surgery, with smaller margins being considered acceptable for preservation of sensitive areas on the vulva and sexual function [15]. Of note, the impact of tumor-free surgical resection margins on survival is addressed only in the German guideline [17].

The aim of this study was therefore to analyze the node-negative patients with SCC of the vulva treated at the Department of Women’s Health, University Clinic, Tuebingen, Germany between 2003 and 2016 with regard to the achieved marginal distance and its impact on prognosis.

## 2. Materials and Methods

This retrospective study was conducted at the Department of Women’s Health, Tuebingen University Clinic, Tuebingen, Germany and included patients who were diagnosed with SCC of the vulva and underwent surgical treatment between 2003 and 2016. Medical charts and pathological reports were reviewed. Patients with SCC of the vulva TNM stage pT1a and higher were included. Documentation and analysis were performed with the support of a Microsoft Excel database (Microsoft Corporation, Redmond, WA, USA). The local ethics committee approved the study protocol (reference number 455/2018BO2).

Initially, a total of 389 patients were documented in the database. The present analysis focused on surgically staged node-negative patients with surgical groin staging, complete tumor resection (R0), and known margin distance (n = 128).

In the database, tumor characteristics as well as aspects of surgical treatment were recorded: TNM stage, tumor size, depth of invasion, grade, type of vulvar surgery, pathological resection margin, type of groin surgery, and date of last contact/death. The clinical cancer registry of the Comprehensive Cancer Center, Tuebingen, Germany provided follow-up and survival data. The review of the pathological reports was “non-blinded”. The patients were subclassified into three resection margin category groups according to the pathological resection margin distance: “1 to 3 mm”, “>3 to 8 mm”, or “>8 mm”. All gross specimens were handled according to the local protocol. All margins of resection were inked. Sections were taken perpendicular to the closest margin in all directions, including the closest deep soft tissue margin, and were documented on a drawing of the specimen. The resection margin distances were determined from tissue sections on hematoxylin- and eosin-stained slides. The closest (minimal) tumor-free margin was determined by taking both the deep tissue (basal) and the peripheral (lateral) pathological margins into consideration. The tumor-free margin was defined as the closest distance from the invasive tumor to the lateral or basal resection margin. The margin refers to the “final” margin after completion of surgical treatment and includes the thickness of additional resections that were taken in the same operative session or during a second surgery.

Surgical treatment of SCC of the vulva consisted either of partial (modified radical) vulvectomy or complete (radical) vulvectomy or of wide excision of the tumor. Lymph node staging consisted of a sentinel lymph node procedure for unifocal tumors < 4 cm with no clinical evidence of lymph node metastasis. For tumors located close to the median line (<1 cm), the sentinel lymph node procedure was performed bilaterally. A systematic inguinofemoral lymphadenectomy was performed in cases of tumors > 4 cm and clinically presumed or pathologically confirmed lymph node metastasis or sentinel lymph node metastasis and before validation of the sentinel lymph node procedure in vulvar cancer. All patients were discussed after the surgical procedure in the multidisciplinary tumor board regarding further therapy options. None of the patients included in the present analysis received (neo-) adjuvant radio(chemo)therapy or systemic therapy. All statistical analyses were performed using R (Version 4.1.1). Patient characteristics were given as median and range or numbers and percentages, as appropriate. Differences between the three resection margin groups were assessed by Kruskal–Wallis tests for continuous variables and by Fisher’s exact tests for nominal data. Disease-free survival (DFS) and overall survival (OS) were analyzed by Kaplan–Meier plots, and groups were compared using the logrank test. To analyze associations between baseline variables and the resection margin, an ANCOVA (analysis of covariance) model was applied. Cox proportional hazard models were used to evaluate the independent factors for survival events. A significance level of 5% was chosen in all tests.

## 3. Results

The study cohort included 128 patients with primary surgically staged node-negative squamous cell cancer of the vulva that underwent surgical treatment at the Department of Women’s Health, University Clinic, Tuebingen, Germany between 2003 and 2016. The patients were subclassified into three resection margin category groups according to the pathological resection margin distance: “1 to 3 mm” (n = 42), “>3 to 8 mm” (n = 47) or “>8 mm” (n = 39). Table 1 shows a summary of the clinicopathological and surgical treatment characteristics for these patients. Median age at diagnosis was 68 years (range: 25–88 years), with the majority of patients having pT1 and pT2 tumors (99.2%) (Table 1). Regarding the types of surgical intervention, most patients (94.5%) received either a partial vulvectomy (60.9%) or a complete vulvectomy (33.6%). The median minimal resection margin in the present cohort of patients was 5 mm (range: 1–11 mm).

Table 2 summarizes the types of lymph node surgery. All patients (100%) underwent an inguinal lymphadenectomy consisting of either sentinel lymphadenectomy, systematic inguinal lymphadenectomy or sentinel-backed systematic inguinal lymphadenectomy (Table 2). A median of six lymph nodes were excised (range: 1–67 lymph nodes) (Table 2). One hundred and ten patients (85.9%) underwent either a sentinel inguinal lymphadenectomy or a sentinel-backed systematic inguinal lymphadenectomy, with 64 patients (50.0%) receiving solely a systematic inguinal lymphadenectomy (Table 2). Additionally, five patients (3.9%) underwent a systematic pelvic lymphadenectomy (Table 2).

Thirty-nine of the 128 patients (30.5%) with squamous cell cancer of the vulva developed recurrent disease after a median follow-up of 6.49 years. Table 3 illustrates the incidence of recurrence dependent on margin distance below and above defined cutoffs (Table 3). Except for depth of tumor invasion, no other factor associated with tumor-free resection margin distance was found (Table 4).

Table 5 summarizes the rates of recurrent disease within the three groups of patients. Disease recurrence occurred in 13 patients (31.0%) with tumor-free resection margin distance of 1 to 3 mm, in 17 patients (36.2%) with tumor-free resection margin distance of >3 to 8 mm and in 9 patients (23.1%) with tumor-free resection margin distance of >8 mm. The rate of recurrent disease showed no statistically significant difference between the three patient groups (*p* = 0.431) (Table 5).

**Figure 1:**
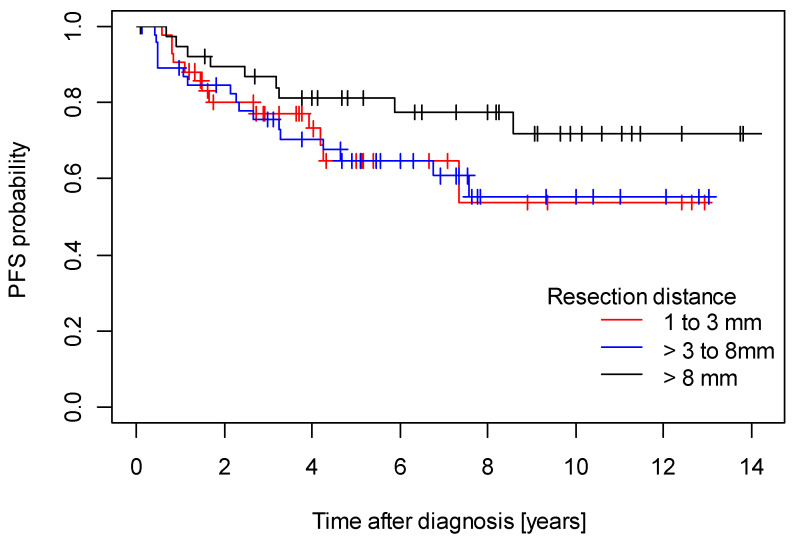
Disease-free survival.

**Figure 2:**
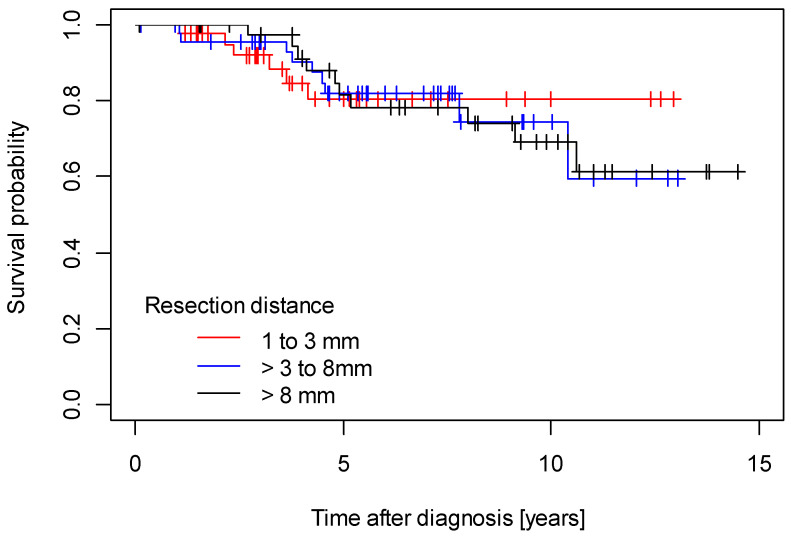
Overall survival.

Median follow-up for disease-free survival was 6.49 years (95% CI 5.16 years; 7.62 years) and median follow-up for overall survival was 6.29 years (95% CI 5.45 years; 6.29 years). The 5-year DFS rate was 0.70 (95% CI: 0.62–0.79), and the 5-year OS rate was 0.79 (95% CI: 0.71–0.87). Regarding the survival outcome, there were no independent significant differences in either disease-free survival (DFS) (*p* = 0.300) or overall survival (*p* = 1.000) among patients within the three tumor-free resection margin categories (1–3 mm, >3 to 8 mm, >8 mm) (Figure 1 and Figure 2).

Multivariate analyses using Cox proportional hazard models did not show any statistically significant association of tumor-free resection margin distance and recurrent disease or death either when analyzed as a categorical variable or when analyzed as a continuous variable (Table 6 and Table 7). Of note, age was a consistent prognostic factor associated with both disease recurrence and death (Table 6 and Table 7).

## 4. Discussion

In this analysis of a large patient cohort with node-negative SCC of the vulva, we analyzed the effects of pathological tumor-free resection margin distance on survival outcome in 128 patients. In our data, we could not find a significant effect of tumor-free resection margin distance on disease-free and overall survival.

Only a few studies to date have addressed the effect of tumor-free resection margin distance on survival in patients with SCC of the vulva. Woelber et al. analyzed 102 patients with SCC of the vulva and suggested that margin distance had no significant influence on disease-free survival after categorizing the patients into three groups (<3 mm, ≥3 mm to 8 mm and ≥8 mm) [11]. However, median follow-up of the analyzed cohort was only 31 months, and almost one-third (30.4%) of the patients received adjuvant radiotherapy [11]. Raimond et al., who also separated the patients into three groups (<3 mm, ≥3 mm to 8 mm and ≥8 mm) analyzed 112 patients with SCC of the vulva and observed no significant impact of tumor-free resection margin distance on disease-free survival and overall survival [18]. However, similarly to the study of Woelber et al., median follow-up of the analyzed cohort was only 25 months and almost one-third (31.2%) of the patients received adjuvant radiotherapy [18]. Viswanathan et al. separated the patients into three groups (positive, <1 cm, ≥1 cm) and found no significant impact of resection margin status on relapse-free survival and overall survival [5]. The median follow-up time was 49.1 months, and 31% of the patients had adjuvant treatment (radiotherapy, radiotherapy and chemotherapy or chemotherapy alone) [5].

Furthermore, Nomura et al. categorized 34 patients with SCC of the vulva into four groups (positive, <3 mm, <5 mm, < 8 mm and ≥8 mm) and noted a significant impact of positive surgical margins and lymph node involvement on recurrence-free survival and overall survival [17]. The median follow-up was 70.5 months and was thus longer compared to the studies by Woelber et al. and Raimond et al. [5,18,19]. Nevertheless, the study population was similarly heterogeneous and included patients receiving either adjuvant chemotherapy or adjuvant radiotherapy for high-risk disease (high initial disease stage, positive resection margins and positive lymph nodes) [19].

Based on these limited and inconsistent results, we reanalyzed the importance of pathological resection margin distance for disease-free and overall survival and found no effect in a large patient cohort with node-negative SCC of the vulva from our institution. As already outlined, most of the existing data on the importance of tumor-free resection margins of SCC of the vulva (with regard to survival) derive from studies that compared heterogeneous groups of patients in terms of risk of recurrence, especially regarding the initial disease extent (patients with initial advanced tumor stage and lymph node involvement) [5,11,18,19]. Consequently, the studies included more than 30% of patients that underwent adjuvant treatment for high-risk disease, thus distorting the effect of surgical resection on disease recurrence and survival. In order to avoid potential confounding factors for long-term prognosis, we chose (similarly to Woelber et al.) to only include patients that underwent surgery for early-stage SCC of the vulva, had node negative-disease and received no (neo-) adjuvant radiotherapy or systemic therapy [20].

In the present study, tumor stage was not significantly different between the three groups of patients. In contrast to Raimond et al., however, the tumor size in our analysis was significantly different between the three groups of patients (*p* = 0.023), with the largest median tumor size in the group of patients in resection margin category 1 to 3 mm [18]. Thus, differences in tumor-free margin distances could be explained by tumor size and initial tumor location. In cases of a large tumor located close to the clitoris, urethra or anus, it is difficult to have a large tumor-free margin without mutilation or dysfunction consequences [18]. Information about whether surgical reconstruction flaps were performed during surgery was not always available in our study, and we chose not to further analyze this aspect. Hence, this provides another possible explanation for observed differences in tumor-free margin distances between patients [18].

Similarly to other studies that had likewise separated patients into three groups (<3 mm, ≥3 mm to 8 mm and ≥8 mm), we did not confirm association between pathological-free margin distance and recurrence risk [5,18,21]. Furthermore, the studies of Woelber et al. and Nooij et al. separated the patients into three groups in a different manner (margin-positive, pathological tumor-free margin < 8 mm, pathological tumor margin ≥8 mm) and found no clear difference in the risk of local recurrence [20,22]. However, not only the pathological-free margin distance but also tumor biology might be a driving factor regarding the risk of recurrence and impaired survival [23,24,25].

The present study has several limitations. The study retrospectively analyzed patients that underwent surgical treatment for SCC of the vulva at a single institution over a 13-year period. During this period, the surgical management of early-stage SCC of the vulva has evolved, mainly through the dissemination of the sentinel node procedure. Furthermore, detailed information regarding the site of recurrence is missing. Additionally, although SCC of the vulva is a disease mostly affecting older patients, information on the cancer-specific mortality rate was not available. Finally, important prognostic categories like HPV association of the tumors were not available for the study at the time of publication but need to be included in further updates of the cohort. Strengths of the study include the large homogenous cohort of node-negative patients with SCC of the vulva that received no adjuvant treatment after surgery and the long follow-up period.

## 5. Conclusions

This study presents the impact of pathological tumor-free margin distance in SCC of the vulva treated with surgery alone on survival, even if the tumor-free margin was less than 2 mm. In the present study, no significant impact of pathological tumor-free resection margin distance on disease-free and overall survival was observed. Thus, a more conservative surgical approach in patients with node-negative SCC of the vulva (leading to less morbidity) might be considered, especially when the objective is preservation of function and sensitive areas on the vulva.

## Figures and Tables

**Table 1:** Patient characteristics.

	Characteristics												
	Age at Diagnosis Years	Tumor Stage			Tumor Diameter mm	Depth of Invasion mm	Grading			Type of Vulvar Surgery			Resection Margin mm
	Median (Range)	pT1 (n, %)	pT2 (n, %)	pT3 (n, %)	Median (Range)	Median (Range)	G1 (n, %)	G2 (n, %)	G3 (n, %)	Wide Excision (n, %)	Partial Vulvectomy (n, %)	Complete Vulvectomy (n, %)	Median (Range)
**Total patients** **(n = 128)**	68 (25–88)	105 (82.1%)	22 (17.2%)	1 (0.8%)	16 (1–95)	2 (1–15)	19 (14.8%)	97 (75.8%)	12 (9.4%)	7 (5.5%)	78 (60.9%)	43 (33.6%)	5 (1–11)
**Resection margin** **category**													
**1 to 3 mm** **(n = 42)**	71 (41–84)	32 (76.2%)	10 (23.8%)	0	22 (1–95)	3 (1–15)	5 (11.9%)	30 (71.4%)	7 (16.7%)	2 (4.8%)	27 (64.3%)	13 (31.0%)	2 (1–3)
**>3 to 8mm** **(n = 47)**	65 (25–88)	41 (87.2%)	5 (10.6%)	1 (2.1%)	17 (1–85)	2 (1–25)	7 (14.9%)	37 (78.7%)	3 (6.4%)	3 (5.6%)	30 (55.6%)	21 (38.9%)	5 ( 4–8)
**>8mm** **(n = 39)**	67 (33–84)	32 (82.1%)	7 (17.9%)	0	10 (1–80)	1 (1–7)	7 (17.9%)	30 (76.9%)	2 (5.1%)	2 (5.1%)	21 (53.8%)	16 (41.0%)	9 (9–11)
***p*-Value**	0.202 ^a^	0.336 ^b^			0.023 ^a^	0.021 ^a^	0.439 ^b^			0.828 ^b^			<0.001 ^a^

^a^ Kruskal–Wallis test. ^b^ Fisher’s exact test.

**Table 2:** Type of lymph node surgery.

	Type of Lymph Node Surgery											
	Inguinal Lymphadenectomy *			Sentinel Lymphadenectomy/ Sentinel-Backed Inguinal Lymphadenectomy			Systematic Inguinal Lymphadenectomy			Systematic Pelvic Lymphadenectomy		
	Yes n (%)	No n (%)	Number of Lymph Nodes: Median (Range)	Yes n (%)	No n (%)	Number of Lymph Nodes: Median (range)	Yes n (%)	No n (%)	Number of Lymph Nodes: Median (Range)	Yes n (%)	No n (%)	Number of Lymph Nodes: Median (Range)
**Total** **(n = 128)**	128 (100%)	0	6 (1–67)	110 (85.9%)	18 (14.0%)	3 (0–14)	64 (50.0%)	64 (50.0%)	0 (0–30)	5 (4.0%)	123 (96.0%)	0 (0–53)
**Resection margin** **category**												
**1 to 3 mm** **(n = 42)**	42 (100%)	0	6 (1–30)	36 (85.7%)	6 (14.2%)	3 (0–14)	19 (45.2%)	23 (54.8%)	0 (0–30)	2 (5.0%)	40 (95.0%)	0 (0–8)
**>3 to 8 mm** **(n = 47)**	47 (100%)	0	6 (1–67)	40 (85.1%)	7 (14.9%)	3 (0–8)	27 (57.4%)	20 (42.5%)	3 (0–26)	2 (4.2%)	45 (95.7%)	0 (0–53)
**>8 mm** **(n = 39)**	39 (100%)	0	6 (1–19)	34 (87.1%)	5 (12.8%)	3 (0–11)	18 (46.1%)	21 (53.8%)	0 (0–19)	1 (2.5%)	38 (97.4%)	0 (0–1)
***p*-value**	1.000 ^b^			1.000 ^b^			0.463 ^b^			1.000 ^b^		
			0.859 ^a^			0.957 ^a^			0.532 ^a^			0.854 ^a^

^a^ Kruskal–Wallis test. ^b^ Fisher’s exact test. * Sentinel lymphadenectomy/systematic inguinal lymphadenectomy/sentinel-backed systematic inguinal lymphadenectomy.

**Table 3:** Incidence of recurrence dependent on margin distance below and above defined cutoffs.

Margin Cutoff	Margin < Cutoff		Margin ≥ Cutoff		*p*-Value (Gray’s Test)	
	N	N Recurrence	N	N Recurrence	Death	Recurrence
2 mm	17	5	111	34	0.280	0.713
3 mm	30	9	98	30	0.916	0.674
4 mm	42	13	86	26	0.476	0.522
5 mm	53	16	75	23	0.947	0.599
6 mm	68	22	60	17	0.428	0.272
7 mm	74	24	54	15	0.516	0.330
8 mm	82	26	46	13	0.240	0.352
9 mm	111	36	17	3	0.156	0.146
10 mm	113	37	15	2	0.090	0.084
11 mm	127	39	1	0	0.716	0.475

**Table 4:** Analysis of covariance (ANCOVA) of the associations between clinicopathological variables and resection margin distance.

Variable		*p*-Value	Coefficient	95 %CI	
**Age (per year)**	continuously	0.304	−0.02	−0.06	0.02
Type of surgery	partial vulvectomy versus wide excision	0.506	0.80	−1.57	3.16
	complete vulvectomy versus wide excision	0.305	1.27	−1.17	3.71
pT	pT2 versus pT1	0.675	0.39	−1.43	2.20
	pT3 versus pT1	0.966	−0.13	−6.13	5.87
Depth of invasion (mm)	continuously	**0.016**	−0.22	−0.41	−0.04
Tumor grade	G2 versus G1	0.784	0.21	−1.32	1.75
	G3 versus G1	0.502	−0.77	−3.03	1.50
Tumor diameter (mm)	continuously	0.323	−0.02	−0.06	0.02

**Table 5:** Disease recurrence in relation to resection margin category.

Characteristics	Total (n = 128)	Resection Margin Category			*p*-Value
		1 to 3 mm (n = 42)	>3 to 8 mm (n = 47)	>8 mm (n = 39)	
**Recurrent disease**					
No	89 69.5%	29 69.0%	30 63.8%	30 76.9%	0.431 ^a^
Yes	39 30.5%	13 31.0%	17 36.2%	9 23.1%	

^a^ Fisher’s exact test.

**Table 6:** Multivariate time-to-event analyses (Cox proportional hazard models) including resection margin as a categorical variable.

Recurrent Disease/Death (50 Events)	Hazard Ratio	*p*-Value	95% CI	
Age [every year]	1.04	**0.003**	1.01	1.07
pT2 versus pT1	0.89	0.791	0.38	2.10
pT3 versus pT1	0.58	0.612	0.07	4.84
Tumor diameter [mm]	1.01	0.247	0.99	1.03
Depth of Invasion [mm]	1.01	0.817	0.92	1.11
Grade 2 versus 1	0.75	0.445	0.35	1.58
Grade 3 versus 1	1.92	0.273	0.60	6.19
Resection margin >3–8 mm versus 1–3 mm	1.26	0.515	0.63	2.53
Resection margin >8 mm versus 1–3 mm	0.93	0.865	0.43	2.04
**Recurrent disease** **(38 events)**	**Hazard Ratio**	***p*-value**	**95% CI**	
Age [per year]	1.04	**0.011**	1.01	1.07
pT2 versus pT1	0.99	0.986	0.37	2.65
pT3 versus pT1	0.00	0.997	0.00	Inf
Tumor diameter [mm]	1.01	0.496	0.98	1.03
Depth of Invasion [mm]	0.97	0.611	0.87	1.08
Grade 2 versus 1	0.82	0.639	0.35	1.91
Grade 3 versus 1	1.28	0.735	0.31	5.22
Resection margin >3–8 mm versus 1–3 mm	1.27	0.533	0.60	2.66
Resection > 8 mm versus 1–3 mm	0.55	0.208	0.22	1.39

**Table 7:** Multivariate time-to-event analyses (Cox proportional hazard models) including resection margin as a continuous variable.

Recurrent Disease/Death (50 events)	Hazard Ratio	*p*-Value	95% CI	
Age [per year]	1.04	**0.004**	1.01	1.07
pT2 versus pT1	0.85	0.705	0.37	1.97
pT3 versus pT1	0.64	0.680	0.08	5.29
Tumor diameter [mm]	1.01	0.238	0.99	1.03
Depth of Invasion [mm]	1.02	0.746	0.93	1.11
Grade 2 versus 1	0.77	0.487	0.36	1.62
Grade 3 versus 1	1.87	0.299	0.57	6.07
Resection margin [mm]	0.99	0.806	0.89	1.09
**Recurrent disease** **(38 events)**	**Hazard Ratio**	***p*-value**	**95% CI**	
Age [per year]	1.04	**0.013**	1.01	1.07
pT2 versus pT1	0.87	0.770	0.33	2.26
pT3 versus pT1	0.00	0.997	0.00	Inf
Tumor diameter [mm]	1.01	0.419	0.99	1.03
Depth of Invasion [mm]	0.98	0.730	0.88	1.09
Grade 2 versus 1	0.86	0.720	0.37	2.00
Grade 3 versus 1	1.23	0.775	0.30	5.10
Resection margin [mm]	0.95	0.318	0.85	1.06

## Data Availability

The data presented in this study are available on request from the corresponding author. The data are not publicly available due to the privacy of sensitive patient data.
